# UNUSUAL WARFARIN DOSE TO ACHIEVE THERAPEUTIC INR IN A 4-MONTH OLD CHILD: NON-GENETICS RISK FACTORS ARE STILL A CHALLENGE

**DOI:** 10.1590/1984-0462/;2017;35;4;00014

**Published:** 2017

**Authors:** Lucas Miyake Okumura, Giovanna Webster Negretto, Clarissa Gutiérrez Carvalho

**Affiliations:** a Hospital de Clínicas de Porto Alegre, Porto Alegre, RS, Brasil.

**Keywords:** Warfarin, Pediatric, Bleeding, Thrombosis, Child, Drug interactions, Varfarina, Pediatria, Sangramento, Trombose, Criança, Interações medicamentosas

## Abstract

**Objective:**

To report a case of a 4-month old girl that required 0.7 mg/kg/day (5 mg) of warfarin and discuss relevant risk factors for requiring higher doses.

**Case Description:**

In November 2015, a 5 kg female infant (36-week preterm) was admitted to the hospital due to status epilepticus and fever. Diazepam, phenytoin and ceftriaxone were prescribed. Cerebrospinal fluid contained 7 leukocytes, 150 mg/dL proteins, 1 mg/dL glucose and gram positive cocci were observed. Cranial tomography suggested hypodense signs in the cerebellum, right temporal lobe and left basal nuclei, which was consistent with pneumococcal meningitis-induced infectious vasculitis. She required low molecular weight heparin and warfarin for post-encephalitis thrombosis. About 10 days were required to achieve therapeutic INR, and warfarin was adjusted five times since the initial prescription.

**Comments:**

The risk factors for higher warfarin doses were age and enteral tube feeding. Phenobarbital and prednisone might also have contributed with one of the highest warfarin dose ever reported. Despite current importance given to genetics testing, clinicians should attempt to identify common contributing factors for prolonged non-therapeutic INR, to minimize the risk of coagulation, and to reduce costs of hospital stay and laboratory exams.

## RESUMO

**Objetivo:**

Descreve se uma criança do sexo feminino, com quatro meses de idade, que necessitou de varfarina 0.7 mg/kg/dia (5 mg). Discutem se os fatores de risco clinicamente relevantes para prescrição de altas doses do anticoagulante em crianças.

**Descrição do caso:**

Em novembro de 2015, uma criança de 5 kg (36 semanas, pré termo) foi admitida no pronto atendimento por *status epilepticus* e febre. Diazepam, fenitoína e ceftriaxona foram prescritos inicialmente. A pesquisa no líquor revelou presença de sete leucócitos, 150 mg/dL de proteínas, 1 mg/dL de glucose e cocos Gram positivos. Em tomografia de crânio, foram observados sinais hipodensos em cerebelo, lobo temporal e núcleos de base à esquerda, sugerindo vasculite infecciosa por meningite pneumocócica. Após o quadro agudo da encefalite, a criança desenvolveu uma trombose e necessitou de anticoagulação com enoxaparina e, posteriormente, com varfarina. Dez dias de tratamento com o segundo fármaco foram necessários para atingir relação normalizada internacional (RNI) terapêutica, tendo sido realizados cinco ajustes de dose desde a primeira prescrição de varfarina.

**Comentários:**

Os fatores de risco determinantes para necessidade de doses maiores de varfarina foram idade e uso de nutrição enteral. Fenobarbital e prednisona também podem ter contribuído para o uso de uma das maiores doses de varfarina já relatadas na literatura. A despeito da importância dos testes de polimorfismo genético, os pediatras devem estar atentos para identificar os fatores que contribuem para a prescrição de maiores doses de varfarina, de forma a minimizar os riscos de trombose, reduzindo os custos com internação e exames laboratoriais.

## INTRODUCTION

Warfarin is a racemic mixture of two active enantiomers (R and S-warfarin) that act by inhibiting vitamin K reductase and reduce the activity and synthesis of clotting factors.^1^ It is widely used for preventing and treating thromboembolic events caused by prosthetic cardiac valves, post-Fontan surgery, portal thrombosis and vasculitis-associated thrombosis in pediatrics.^2^ Infants require 0.33±0.2 mg/kg/day of warfarin to achieve therapeutic International Normalized Ratio (INR).^3^

This study aimed to report a case of a 4-month old girl that required warfarin 0.7 mg/kg/day, and discuss relevant risk factors for requiring higher doses.

## CASE DESCRIPTION

In November 2015, a 5kg female infant (36-week preterm) was admitted to the hospital due to status epilepticus and fever. Diazepam, phenytoin and ceftriaxone were prescribed. Cerebrospinal fluid contained 7 leukocytes, 150 mg/dL proteins, 1 mg/dL glucose, and gram positive cocci were observed. Cranial tomography suggested hypodense signs in the cerebellum, right temporal lobe and left basal nuclei, which was consistent with pneumococcal meningitis-induced infectious vasculitis. Other relevant medical history included HIV vertical exposure (4 sequential undetectable viral loads during follow-up).

She stayed in the critical care unit for 18 days until support therapy was no longer necessary. By the time she was discharged to the pediatric ward, she was on enteral nutrition, prednisone taper after high dose dexamethasone, phenobarbital 5 mg/kg/day and enoxaparin (LMWH) 1 mg/kg q12h, for post-meningoencephalitis thrombosis.

**Figure 1:**
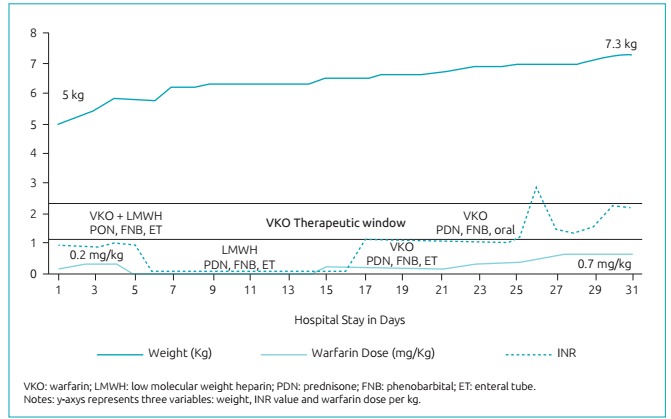
Warfarin dose requirements and INR.

On December 15^th^, warfarin 0.2 mg/kg/day was bridged with LMWH and adjusted according to INR (Figure 1). From Day 21-23, she received 0.3 mg/kg of warfarin, but INR remained unchanged. On day 24, she was allowed to eat (supplements, as per dietician’s prescriptions) and receive medications per oral route, when INR started to increase until therapeutic levels. From day 27-30 she received 0.7 mg/kg of warfarin and was discharged with INR=2.3, after clinical pharmacist’s education session. In summary, about 10 days were required to achieve therapeutic INR, and warfarin was adjusted five times, since initial dosing.

## DISCUSSION

Previous reports^3^ ^,^ ^4^ ^,^ ^5^ ^,^ ^6^illustrate the influence of isolated risk factors on high warfarin dose requirements. None of these reports demonstrated the synergistic influence of multiple risk factors resulting in higher doses or multiple adjustments in multivariate analysis.

In regards to drug-drug interaction, phenobarbital could have contributed with the highest warfarin dose ever reported, since it is known the drug interaction on CYP as enzyme inhibitor mechanisms.^3^ Paradoxically, the use of corticosteroids could contribute to lower warfarin dose requirements to achieve therapeutic INR levels, as previously seen in patients with CYP2C9 polymorphisms.^7^ However, in the present case, the use of prednisone and dose tapering strategy did not significantly affect INR and warfarin dose changes (Figure 1).

The striking risk factors for higher warfarin doses were age and enteral tube feeding. Age-related factors, such as infants^3^ need higher doses of warfarin (~0.3 mg/kg) than older children (0.08 mg/kg). The exact mechanism of such discrepancy still is not elucidated, but may be related^4^ to liver development.

Administering warfarin through enteral tubes has been associated with higher warfarin doses (0.28 mg/kg) in comparison^3^ to children who are not on enteral feeding (0.16 mg/kg). In the present case, INR achieved therapeutic levels after removal of the enteral tube (day 24). It is noteworthy that, before day 23, a speech pathologist was consulted to verify the possibility to progress from enteral tube to oral diet, which consisted in supplement-based nutrition as per dietician’s prescription (breastfeeding was not allowed due to the HIV vertical transmission). In this case, the child received oral stimulation interventions and the risk of aspiration was excluded, before initiating oral nutrition.

Notably, controls (thrombin time) were always the same during hospital stay (12.2s), and patient was not eligible for coagulopathy investigation, as infectious vasculitis was the primary cause of thrombosis. Additionally, this patient could be a candidate for VKORC1 and CYP2C9 polymorphisms assessments, as they are associated with different warfarin dosing requirements.^6^ However, such exam is not routinely made in our institution and more studies are required in children.^6^ ^,^ ^8^ It is worth noting that VKORC and CYP2C9 gene polymorphism screening have been issued as an essential laboratory test before initiating warfarin.^9^ ^,^ ^10^ While the clinical value of such exam has been associated with improved safety,^9^ ^,^ ^10^ the cost-effectiveness^11^ of universal testing of patients initiating vitamin K antagonists still is to be determined, especially in public health systems. A randomized trial^11^ of routine VKORC and CYP2C9 polymorphism testing will also analyze how cost-effective is such technology in Brazil, in order to assess the priority of such pharmacogenetics information.

Clinicians should attempt to identify contributing factors for prolonged non-therapeutic INR, to minimize the risk of coagulation, reducing costs with hospital stay and laboratory exams. While warfarin-related polymorphism testing is gaining space in pediatrics, based on the presented case report, evaluation of non-genetics risk factors is still essential and potentially manageable. Other effective measures for preventing adverse events related to warfarin therapy could also include integrated inpatient and outpatient anticoagulation services and the use of computerized physician order entry systems with alerts to guarantee that patients will achieve the desired levels anticoagulation effects.^12^ ^,^ ^13^

## References

[1] Holford NH (1986). Clinical pharmacokinetics and pharmacodynamics of warfarin. Understanding the dose-effect relationship. Clin Pharmacokinet.

[2] Monagle P, Chan AK, Goldenberg NA, Ichord RN, Journeycake JM, Nowak-Göttl U (2012). Antithrombotic therapy in neonates and children. Chest.

[3] Streif W, Andrew M, Marzinotto V, Massicotte P, Chan AK, Julian JA (1999). Analysis of warfarin therapy in pediatric patients: A prospective cohort study of 319 patients. Blood.

[4] Takahashi H, Ishikawa S, Nomoto S, Nishigaki Y, Ando F, Kashima T (2000). Developmental changes in pharmacokinetics and pharmacodynamics of warfarin enantiomers in Japanese children. Clin Pharmacol Ther.

[5] Wells PS, Holbrook AM, Crowther NR, Hirsh J (1994). Interactions of warfarin with drugs and food. Ann Intern Med.

[6] Zhang J, Tian L, Zhang Y, Shen J (2015). The influence of VKORC1 gene polymorphism on warfarin maintenance dosage in pediatric patients: A systematic review and meta-analysis. Thromb Res.

[7] Ruud E, Holmstrøm H, Bergan S, Wesenberg F (2008). Oral anticoagulation with warfarin is significantly influenced by steroids and CYP2C9 polymorphisms in children with cancer. Pediatr Blood Cancer.

[8] Moreau C, Bajolle F, Siguret V, Loriot MA, Bonnet D (2013). Genetic resistance to warfarin therapy masked by amiodarone in a 2-year-old girl with mitral valve replacement. J Thromb Haemost.

[9] Johnson JA, Gong L, Whirl-Carrillo M, Gage BF, Scott SA, Stein CM (2011). Clinical Pharmacogenetics Implementation. Consortium Clinical Pharmacogenetics Implementation Consortium Guidelines for CYP2C9 and VKORC1 genotypes and warfarin dosing. Clin Pharmacol Ther.

[10] Shaw K, Amstutz U, Kim RB, Lesko LJ, Turgeon J, CPNDS Clinical Recommendation Group (2015). Clinical Practice Recommendations on Genetic Testing of CYP2C9 and VKORC1 Variants in Warfarin Therapy. Ther Drug Monit.

[11] Marcatto LR, Sacilotto L, Bueno CT, Facin M, Strunz CM, Darrieux FC (2016). Evaluation of a pharmacogenetic-based warfarin dosing algorithm in patients with low time in therapeutic range - study protocol for a randomized controlled trial. BMC Cardiovasc Disord.

[12] Jones S, McLoughlin S, Piovesan D, Savoia H, Monagle P, Newall F (2016). Safety and Efficacy Outcomes of Home and Hospital Warfarin Management Within a Pediatric Anticoagulation Clinic. J Pediatr Hematol Oncol.

[13] Miller AM, Boro MS, Korman NE, Davoren JB (2011). Provider and pharmacist responses to warfarin drug-drug interaction alerts: a study of healthcare downstream of CPOE alerts. J Am Med Inform Assoc.

